# Supplementary figures and images for: Leprosy in skeletons from archaeological sites: A systematic review
Source: PLoS Negl Trop Dis. 2025 Aug 19;19(8):e0013374. doi: 10.1371/journal.pntd.0013374 (PMC12364322; doi:10.1371/journal.pntd.0013374)

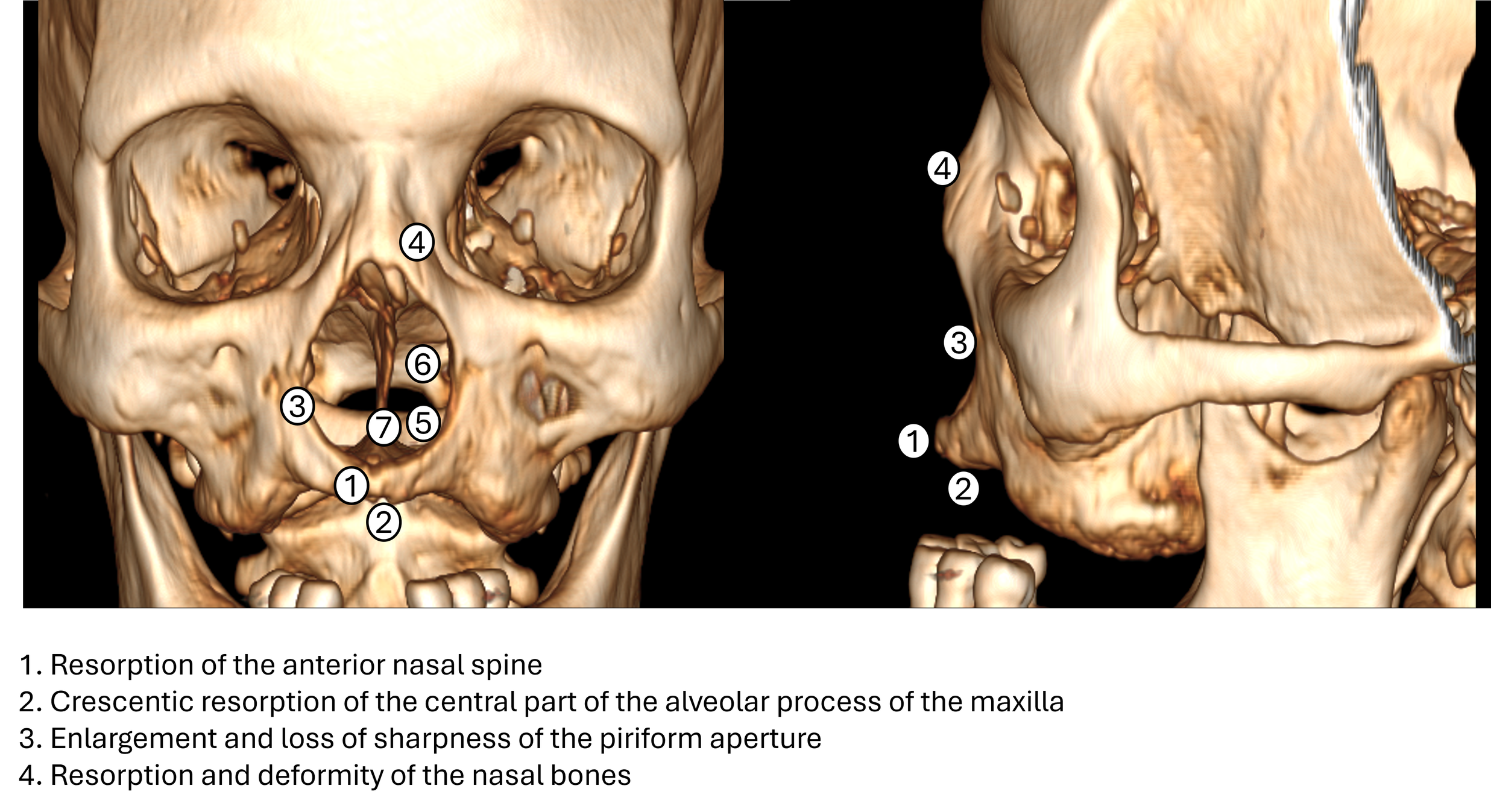

Supplement: S1 Fig — (TIF) [file pntd.0013374.s001.tif]
