# Supplementary material for: Leprosy in skeletons from archaeological sites: A systematic review
Source: PLoS Negl Trop Dis. 2025 Aug 19;19(8):e0013374. doi: 10.1371/journal.pntd.0013374 (PMC12364322; doi:10.1371/journal.pntd.0013374)
Supplement: Attachment — Submitted filename: Responses to Reviewers.docx [file pntd.0013374.s006.docx]

**Response to Reviewers’ Comments**

**A. Methods**

Reviewer #1: The aims and methods and approaches taken are clearly outlined. The MS is a revue article of the palaeopathology and phylogeography of leprosy due to M. leprae through time and as such is ambitious. It is necessarily governed by several pre-existing limitations; the archaeological record available Genomic studies are further restricted by permissions for destructive sampling, DNA survival and current aDNA techniques obtaining at their time of study.

**We appreciate the reviewer recognizing the ambition of our review and for their incisive and insightful comments. We have attempted to address these in full, and we think that the revised manuscript is much improved. We have added to our discussion of limitations further points around the archaeological record and the specific point of ‘ethical and legal constraints surrounding destructive sampling’.**

Reviewer #2: The objectives of the study are clearly articulated with clearly testable hypothesis.
The systematic review is well-defined. The only critical point is that there is a discrepancy between the 86 in the systematic review and the overall 134 references. 40% of the references are not accounted for. The authors should mention that the other set of references are included on the basis of snow-balling or another approach.

**We thank the reviewer for their encouraging remarks and for insightful comments, which we have attempted to address in full. Regarding references, the first 39 were cited in the Introduction and Methods, followed by references to the 86 included studies, plus 9 further citations to support our Discussion, yielding 134 references in total. In revising our manuscript, we have added two more references to the Introduction and eleven more to the Discussion in response to reviewers’ comments. Note that Methods is now final section hence, all references have been re-ordered.**

Reviewer #3: There needs to be a clearer objective complete with hypothesis for testing. This will improve the study design, structure of results and study design. The authors also need to consider whether they have enough material to support some of their conclusions, e.g. you can't talk about the prevalence of a strain of a disease when n is low or even 1. The statistical analysis for what they did was fine, but it lacks detail and is very basic. In addition, there has been limited engagement with the limitations of palaeopathological data which impacts on the soundness of the conclusions.

**We thank the reviewer for their critical review of our study and its reporting. We have attempted to address their incisive and insightful comments in full, and we think that the revised manuscript is much improved. Our stated primary objective (Introduction, final paragraph) was “to characterize the frequency of occurrence of leprosy-related bone changes reported in archaeological studies, quantifying as primary outcomes the proportion of human remains in which probable or possible RMS and/or post-cranial alterations could be identified” and our secondary objectives were “to describe variation in RMS over time in Europe, to investigate correlations of RMS with post-cranial alterations, to describe frequency of bone alterations by age group, and to summarize *M. leprae* genotypes identified from ancient DNA.” Hence, all of our objectives were to describe (summarize) the paleopathological data using methods (formal systematic review) that have not been applied previously to this subject. In investigating variation over time and correlations, we are not testing pre-specified hypotheses, and the nature of the data (cross-tabulations of frequencies) are such that the appropriate statistical tests are necessarily basic. We have made a large number of revisions, as described below, including engaging in more detail with the limitations (and potential modern day utility) of paleopathological data, and we think that these have greatly improved the manuscript. We have replaced the term ‘prevalent’ (with ‘more frequent’) where this might be misconstrued as having an epidemiological (population-level) meaning.**

**B. Results**

Reviewer #1: The Results are clearly presented in the main paper with Tables, flow diagrams and Figures. This is supplemented by a great deal of additional and detailed information in the Appendices and supplementary Tables in excel spreadsheet format. There are some inconsistencies and gaps in the latter section which could be corrected, and specific suggestions have been made in the main review for the authors' consideration. See Summary and General Comments below.

**No comments to address.**

Reviewer #2: The results are clearly and completely represented

**No comments to address.**

Reviewer #3: Generally, yes, but the aims and objectives are rather loose. The results are sometimes incomplete. There are no statistical tests results presented in the text and it is unclear when they were used. In addition, there is a lack of detail about whether correction factors were used or not. When presenting skeletal data we are not always presented with observation values, the hand and foot data is particularly problematic and it is hard to know whether it is meaningful or not. There is use of vague terminology (see comments below).

**See response to previous comment regarding aims and objectives. We have tightened up our reporting where percentages were reported and statistical tests were performed, adding the fraction from which the % was calculated and including p-values in the text where any differences were described, adding the caveat where appropriate that “small numbers of bones were available for analysis and we performed multiple statistical tests”.**

**C. Conclusions**

Reviewer #1: (No Response)

**No comments to address.**

Reviewer #2: The conclusions are concise and well-structured.

**No comments to address.**

Reviewer #3: The conclusions are weak really since the data is a little problematic (see comments below). The limitations of the study are not adequately addressed and there is almost no engagement with the issues surrounding the use of palaeopathological data and its limitations. At present, there is very limited advancement of knowledge as many of the issues raised relate to information that is present in the cases that they have drawn together rather than new findings identified by the authors in the results. Where they have drawn new conclusions surrounding the lesion patterns, these are weak as they have not considered the biased nature of the sample they are using (already preselected for leprosy and the most severe forms at that) and the fact that they discarded cases that were not characteristic.

**The aim of our review was to draw together and describe paleopathological data presented in published studies that we identified systematically, i.e., thoroughly and in a way that should be replicable (using systematic review methodology typically applied to medical literature). We believe that the body of information thereby generated is of use as an aggregate data source, even if our review has not advanced knowledge (because it is using secondary data rather than generating primary data). We have expanded upon the limitations of our source data and we have re-written our conclusion to align with the aims and design (and limitations) of our review.**

**D. Editorial and Data Presentation Modifications**

**Abstract**

The abstract does not make it clear why the research is important or needed, or moves beyond what is already known.

**We have re-written our abstract within the word count constraints, including a more focused statement of our aim.**

**Introduction and methods**

Just a note, Hansen’s Disease has its own problems since Hansen himself had some pretty unorthodox and unethical practices (<https://worldneurologyonline.com/article/armauer-hansen-the-controversy-surrounding-his-unethical-human-to-human-leprosy-transmission-experiment/>).

**Indeed, we are aware of this nominative conundrum and have published on it (Deps P, Cruz A. Why we should stop using the word leprosy. Lancet Infect Dis. 2020 Apr;20(4):e75-e78. doi: 10.1016/S1473-3099(20)30061-X). Whilst our clinical publications tend to use the term Hansen’s, we have used leprosy in this manuscript because this is still widely used in paleopathological literature.**

Page 3 Reference needed for incubation time

**Citation added: Pinheiro RO, de Souza Salles J, Sarno EN, Sampaio EP. Mycobacterium leprae-host-cell interactions and genetic determinants in leprosy: an overview. Future Microbiol. 2011 Feb;6(2):217-30. doi: 10.2217/fmb.10.173.**

Reference for MB being the cause of most transmissions

**Citation added: Pinheiro RO, de Souza Salles J, Sarno EN, Sampaio EP. Mycobacterium leprae-host-cell interactions and genetic determinants in leprosy: an overview. Future Microbiol. 2011 Feb;6(2):217-30. doi: 10.2217/fmb.10.173.**

Page 4 Leprosy was not eliminated from Europe by the 16th century, only parts of it. It was still found in Scandinavia, Iberia and some Eastern Areas for a long time after this.

**Amended to ‘largely eliminated by the 18^th^ Century’.**

How would changes in population density impact leprosy? Are you suggesting a link to the plague mortalities here?

**This sentence amended and mention of population density removed: ‘As seen in the modern era, improved living conditions (23–25) and better nutrition (26,27) may have also contributed to this decline’.**

In reference to citation 31. Is this 3-5% of modern patients?

**Yes, clarified by amending to ‘3-5% of clinical cases’.**

P5 Criteria for inclusion and scoring. Where can we find the information about how these scores are defined.

**Clarification added that the scoring system had 8 criteria (items), or 6 for genomic studies, and that information about the scores can be found in Supporting Information Appendices.**

Why did you exclude Eurasian countries in Europe? What is your definition here, since Europe is a fairly modern construct. What about Belarus?

**Our review was motivated by interest in the historical distribution, presentation and decline of leprosy in what we term ‘modern-day Europe’ hence, excluding Eurasian countries and including western, northern, central/eastern and southern European countries. We recognize that there might be other definitions of ‘modern-day Europe’. We have corrected our summary of genomic studies to include Belarus in our definition.**

P6 – unless there are burial records we can not assign year of death, only a range.

**Data extraction method amended to ‘year of death (from burial record) else range’.**

In table headings, put references of the work that you are deriving your information from.

**Table 1 contains references to specific studies in the body of the table. The other tables present summary data from a large number of studies, as cited in the first part of our Results section (under ‘Geographic and demographic data (non-genomic studies)’). We feel that citing all of the references in the table heading is unnecessary and we have instead inserted the number of studies from which the information in the table was derived, e.g., ‘Table 2. Frequency of skeletons with leprosy (N=297, from 67 studies) showing sex, age at death and type of burial site’**

In data analysis, please let us know the program used for the tests, in addition please state whether you used correction factors for a) small sample sizes, and b) multiple testing (if appropriate).

**Methods ‘Data analysis’ expanded to include statistical software citation, namely: ‘Differences in frequencies of bone alterations by the presence or absence of RMS and by age group were tested in Stata (StataCorp. 2023. Stata Statistical Software: Release 18. College Station, TX: StataCorp LLC.) using Fisher’s exact test (alpha=0.05)’. Use of Fisher’s exact test indicates correction for small sample size; α=0.05 indicates no correction for multiple testing.**

**Results**

Make sure that before you present any %s you give the numbers observable.

**We have corrected these omissions throughout the manuscript.**

Page 10, you don’t talk about the child cases that you discuss later. They are also not in the table. (page 12).

**We have added rows for the child cases to Table 2 with a short paragraph beneath giving more details of ages. We have also clarified in text and footnote that the age is the mid-point of the estimated age range in the absence of a burial record.**

Data presentation. Please always let us know how many observations there were before providing any statistics (by individual or bone etc). This is critical so that we know how meaningful any percentages are. For example ‘Varying numbers of remains had data for specific cranial bones’ – very vague. In addition, please provide test statistic results (e.g. n values, test statistic and p Value).

**We have corrected these omissions throughout the manuscript and we have re-worded the specific sentence mentioned, to ‘The number of skeletons that provided data for specific cranial bones ranged from n=45 for posterior alveolar margins of the maxilla to n=279 for maxilla (of N=297 skeletons)’.**

Use of the term ‘ante mortem’. This could refer to any change on the skeleton before death, pathology, trauma, behavioural, activity related etc. I would say pathological changes so it is clear you are looking at disease-related change.

**We have changed the term ‘ante mortem’ to ‘pathological’ throughout the manuscript.**

Page 13 You mention differences in cranial changes by age but you don’t say what you see in the data.

**We have added a description, namely “a higher proportion with irregular perforation of the palate in the ≥50 years age group (80%, 8/10) compared with 37.8% (17/45) and 32.3% (20/62) in the 0-24 and 25-49 years age groups, respectively”.**

Nasal structures. Second sentence is incomplete?

**Corrected.**

P15 RMS – Tell us why you are referring to dental abscess data.

**We have added a text summary of the dental abscess data in a separate section. The reason for reporting these data in relation to leprosy is that leprosy-related oral lesions can create an environment conducive to bacterial infection, potentially leading to dental abscesses. We have added this explanation to our Introduction, citing Dave B, Bedi R. Leprosy and its dental management guidelines. Int Dent J. 2020 Nov 5;63(2):65–71.**

The data for the hands and feet is confusing. It would probably be more useful to present this by individual since there was likely high variability in the recovery of tarsals by individual. I know that Winchester had excellent recovery because they a) knew they had leprosy so explicitly focused on recovering hand and foot bones b) had properly train osteologists working on site. Whereas isolated cases in cemeteries might not have been looking for everything. As such, your numbers could be biased by differences in excavation practice.

**We have added this as another limitation of our study, namely “We presented post-cranial bone changes in aggregate, but significant variability likely exists in the recovery and documentation of hand and foot bones, influenced by the specific objectives of archaeological excavations and the availability of osteological expertise at different sites.”**

P16 The increasing prevalence of inflammation (periostitis, osteomyelitis etc) with age is found in palaeopathology generally regardless of leprosy. Simply, the older you are, the more time you have for accumulating bone changes.

**We have added this remark to our reporting of the results, namely “although numbers are small and apparent differences unsupported by statistical evidence (p=0.140) (Table S9); increasing prevalence of inflammation with age is also likely regardless of leprosy.”**

P18 – I am not sure about the title ‘ skeletons from modern day Europe’. Should this be skeletons from the region of modern day Europe?

**Corrected, also one other occurrence in first paragraph of Discussion.**

**Discussion**

In the first paragraph of the discussion, the authors highlight the relevance of the topic to modern problems posed by the disease, but they don’t say how it can be used specifically. What has this specific piece of work contributed to these wider questions?

**We have extended this paragraph to provide more detail as to how “Direct examination of bone changes in paleopathology specimens can improve understanding of clinical manifestations of *M. leprae* infection in living persons affected by Hansen’s disease (122). For example, we previously used computed tomography (CT) scans to assess the prevalence of RMS among elderly former patients of a ‘colony’ hospital in Brazil (123). We also used CT imaging combined with otorhinolaryngological examinations to assess maxillofacial and nasal manifestations of leprosy in current patients (124). Findings from these studies suggest that clinical protocols for Hansen’s disease could be extended to include otorhinolaryngological evaluation, supported by imaging where necessary, to improve the assessment and overall care of persons affected by Hansen’s disease. This systematic review synthesizes evidence from palaeopathological studies, characterising cranial and post-cranial bone alterations associated with leprosy, serving as a reference point for clinical research and practice today.” supported by references:**

**122. Deps P, do Espírito Santo RB, Charlier P, Collin SM. Rhinomaxillary syndrome in Hansen’s disease: a clinical perspective. Int J Dermatol. 2020 Nov;59(11):e404–6.**

**123. do Espírito Santo RB, Serafim RA, Loureiro RM, Sumi DV, de Mello RAF, Nascimento IF, et al. Clinical and radiological assessment of rhinomaxillary syndrome in Hansen’s disease. Indian J Dermatol Venereol Leprol. 2022;88(4):483–93.**

**124. do Espírito Santo RB, Serafim RA, Loureiro RM, Gonçalves DVC, Sumi DV, de Mello RAF, et al. Clinical and radiological evaluation of maxillofacial and otorhinolaryngological manifestations of Hansen’s disease. Sci Rep. 2022 Sep 1;12(1):14912.**

**We have also extended our ‘Leprosy outside Europe’ section with a discussion of how evidence from paleopathological research can inform One Health approaches to the control of Hansen’s disease in the present day (in response to another review comment).**

In terms of understanding *M. leprae* evolution, you do not discuss how the different strains are related to each other genetically and temporally. Also, I am not sure we can say which strains were prevalent for most geographic areas because for most places there are one or two cases. Only really for the UK or Denmark where multiple cases are analysed, and this would only be true for one period. There is no real new information presented here, rather just a repeat of what is known and presented directly in the genetic studies. For example, Monot et al 2009, Mark 2017, Schuenemann et al 2018, Pfrengle et al 2021.

**We have completely re-written our discussion of results from genomic studies, addressing points made about genomic data in this and related comments and referring readers to the work of Schuenemann and Pfrengle, specifically:**

**Schuenemann VJ, Avanzi C, Krause-Kyora B, Seitz A, Herbig A, Inskip S, et al. Ancient genomes reveal a high diversity of Mycobacterium leprae in med ieval Europe. PLOS Pathog. 2018 May 10;14(5):e1006997.**

**Pfrengle S, Neukamm J, Guellil M, Keller M, Molak M, Avanzi C, et al. Mycobacterium leprae diversity and population dynamics in medieval Europe from novel ancient genomes. BMC Biol. 2021 Oct 5;19(1):220.**

Page 23 – method for analysing the skulls from images. How many were done like this? Please put detail in the methods section for this. How reliable is this likely to be? Any intra observer tests for this?

**We have clarified (in Methods and Discussion) that presence/absence of RMS was determined from extracted data only (not by inspection of images in included references).**

P24 paragraph 3 ‘According to Moller Christensen, cranial bone changes associated with leprosy deformities in the hand and feet result in nasal alterations’ This does not make sense I am afraid. Rephrase.

**Corrected to “cranial bone changes associated with leprosy were strongly correlated within nasal alterations, such as..”**

You state that the proportion of RMS is higher in the 16th century, but is this due to them living longer, and the fact they are all from leprosaria (see above). Number of cases is also very low.

**We agree, and had put forward these reasons in our Discussion, to which we have added a comment about the number of cases, namely: “Our review suggested more frequent RMS in the relatively small number of remains from the 16^th^ century onwards, but this was unsupported by statistical evidence and may reflect the state of preservation of remains and increased longevity (hence, more time for disease to progress)”.**

P25 Can you just outline why you are presenting the specific cases you are looking at them?

**These specific cases were selected because they represented either the oldest (time period) specimens or the first to undergo genomic testing. We have moved this text from the Discussion to the corresponding subsections in Results, namely “The oldest skeleton diagnosed with leprosy..” to the ‘RMS’ subsection and “..the oldest to test positive for *M. leprae*..” to the ‘Genomic studies’ subsection.**

P25 – these cases are not described in your table previously, no children or babies are presented there. Were they excluded cases? This is the first time they are mentioned and as such the inclusion in the discussion is a bit random and doesn’t relate to any of the presented results.

**We have added rows for the child cases to Table 2 with a short paragraph beneath giving more details of ages. We have also clarified in text and footnote that the age is the mid-point of the estimated age range in the absence of a burial record.**

In relation to the decline of leprosy, it is interesting that the lesion pattern is similar to S Korea, but a bit of caution is needed here, since a similar change could also be seen if there were improvements in care, or changes in care that allowed people to live longer with the disease. In addition, you did not find any clear statistical relationship between age and lesions. It is likely that the sample sizes are just too small to do this.

**We have removed the remark about the trend in S Korea being ‘consistent with a decrease in infection transmission in recent decades’ and we have added a comment about the lack of statistical evidence and small number of cases, namely: “Our review suggested more frequent RMS in the relatively small number of remains from the 16^th^ century onwards, but this was unsupported by statistical evidence and may reflect the state of preservation of remains and increased longevity (hence, more time for disease to progress)”.**

Top of page 27 In the UK, France, Spain and Belgium, ideas about the historic isolation of individuals with leprosy have been dismissed. Such isolation narratives have been particularly harmful to those with the disease today and are proven false. Please change the wording.

**We are very cognisant of latter day practices leading to harmful narratives in the modern era, and our sentence was simply stating that leprosaria and related practices became more common from the 11^th^ Century onwards. However, we have deleted the part of the sentence that referred to isolation practices in case this is misconstrued as the authors being supportive of these which, coming from an endemic country, we are definitely not.**

P28 – You cannot use the high prevalence of nasal changes from a group of individuals that are identified because they have nasal changes as evidence for their importance in diagnosis. This is circular reasoning.

**We have deleted these two sentences.**

Yes, I totally agree that there is significant variation in the prevalence and type of bone alterations due to site selection, geography etc etc. Please can you discuss this in more detail.

**We have added some detail to our Discussion as follows: “Although the location and type of archaeological site might be expected to influence the types of bone alterations in remains, we found equal proportions (15%) of skulls without RMS from leprosaria and from general cemeteries or other burial sites. This observation challenges the assumption that leprosaria served as focal destinations and final resting places for people in advanced disease stages, who would presumably exhibit distinctly identifiable facial deformities (122,123) or that individuals within leprosaria necessarily experienced prolonged survival, allowing more time for such deformities to develop. However, we also recognise that the entire paleopathological population in our review is pre-selected for the more severe forms of leprosy that result in visible bone changes, regardless of site of exhumation, which explains the overall high frequency of RMS in our study.” citing:**

**122. Deps P, do Espírito Santo RB, Charlier P, Collin SM. Rhinomaxillary syndrome in Hansen’s disease: a clinical perspective. Int J Dermatol. 2020 Nov;59(11):e404–6.**

**123. do Espírito Santo RB, Serafim RA, Loureiro RM, Sumi DV, de Mello RAF, Nascimento IF, et al. Clinical and radiological assessment of rhinomaxillary syndrome in Hansen’s disease. Indian J Dermatol Venereol Leprol. 2022;88(4):483–93.**

People are undertaking NGS and WGS in archaeology and people are doing interdisciplinary papers. In particular there are multiple papers that combine genetics, geochemistry, pathology and dating. There are also a couple of papers looking at the role of non-human hosts (armadillos, squirrels). This latter is particularly important in modern contexts where animals are not considered as possible reservoirs for the disease.

**We have added some discussion on One Health to address the latter point, namely: “One aspect of paleopathological research of relevance to the present day is the role of animal and environmental reservoirs of *M. leprae* (and *M. lepromatosis*) and the potential for zoonotic transmission to introduce and sustain infection in human populations (143). In present day South America, particularly in Brazil where Hansen’s disease remains endemic, *M. leprae* is prevalent in armadillos, and human contact through hunting and consumption is common, there is a compelling argument for adopting a One Health approach to Hansen’s disease (144), whilst further evidence is needed to assess the zoonotic potential of *M. lepromatosis* in the region (145). Genetic evidence from medieval Europe reinforces these links, showing close relationships between ancient *M. leprae* strains from Iberia and England and those isolated from modern red squirrels. Moreover, strains from the Late Medieval period are genetically similar to those now circulating in humans and armadillos in the Americas (146). These findings underscore how palaeopathological research clarifies historical transmission patterns that remain relevant today, supporting integrated, cross-disciplinary strategies to control leprosy in endemic settings” citing:**

**143. Ploemacher T, Faber WR, Menke H, Rutten V, Pieters T. Reservoirs and transmission routes of leprosy; A systematic review. PLoS Negl Trop Dis. 2020 Apr;14(4):e0008276.**

**144. Deps P, Rosa PS. One Health and Hansen’s disease in Brazil. PLoS Negl Trop Dis. 2021 May 27;15(5):e0009398.**

**145. Deps P, Collin SM. Mycobacterium lepromatosis as a Second Agent of Hansen’s Disease. Front Microbiol. 2021 Sep 10;12:698588.**

**146. Pfrengle S, Neukamm J, Guellil M, Keller M, Molak M, Avanzi C, et al. Mycobacterium leprae diversity and population dynamics in medieval Europe from novel ancient genomes. BMC Biol. 2021 Oct 5;19(1):220.**

**E. Summary and General Comments**

**Reviewer #1**

A). Leprosy main paper:

The authors have reviewed a large number of studies (n=67) dealing with both the palaeopathology and biomolecular lineages of M. leprae cases isolated from antiquity to the post medieval period (3715 BCE to 1839 CE). A total of 297 skeletons with leprosy have been reviewed and the ontological data compiled and correlated by age, sex, period and various other categories. The skeletal patterning due to leprosy has been much studied in the modern era since the late 1950’s, so osteological signs of damage due to M. leprae and secondary infections in the extremities of the appendicular skeleton and their associations with rhinomaxillary syndrome are well recognized. Consequently, many of the findings in the current review are not unexpected. The submission emphasizes the importance of combining paleopathological and biomolecular approaches leprosy but does not highlight specific areas for future study.

**We have revised our conclusion to give some pointers for future research, namely: “The integration of genetics and, potentially, biomolecular approaches such as those based on mycolipids, proteins and peptides into paleopathology, will be essential for validating the diagnoses in archaeological specimens and for elucidating the complex interactions between *M. leprae* and human populations throughout history.” We have also suggested that “Newer imaging modalities such as micro-CT may allow for deeper characterisation of bone alterations and provide a way of confirming leprosy diagnosis in fragmented remains (147). These techniques may also support age-matched comparisons between individuals from leprosaria and those from general burial sites, enabling researchers to explore the effects of institutional care, treatment access, and disease progression on skeletal pathology.” citing:**

**147. Robbins Schug G, Mahajan S, Carter S, Leach A, Williams KD, Douglas KA, et al. Lepromatous leprosy in Bronze Age Oman: micro-CT provides tools for paleopathology in fragmentary and commingled assemblages. Front Med. 2025;12:1521515.**

It is appreciated that this undertaking represents a great deal of data mining and assessment from a large body of work. The review is very comprehensive but does not mention one of the rarer manifestations of multibacillary leprosy sometimes seen in adolescent remains, leprogenic odontodysplasia (LO) (Danielsen K. Tandlaegebladet 1970; 74:603–625). The phenomena has only been described in the palaeopathological literature and invariably from North-Western Europe. The current submission cites this in included reference article 39 (Matos & Santos. 2013) and in the main article (ref 46) but does not record detail. LO has also been seen in 4 individuals from the St. Mary Magdalen leprosaria in Winchester, Hants. UK (article ID 66 and main text ref. 59). For completion, the appropriate place to record this would be the Data Extraction Table alongside the other dental pathology.

**We thank the reviewer for this suggestion. Rather than make post hoc changes to our data extraction, we have instead added the following paragraph to our Discussion of ‘Cases of leprosy in childhood’: “*Leprogenic odontodysplasia* is a manifestation of multibacillary leprosy that affects the normal development of tooth roots, particularly of the maxillary incisors, in conjunction with RMS, described first by Danielsen in the skeletons of four children aged 8-11 years from the medieval leprosy cemetery at Naestved, Denmark (134). There are no records of clinical cases. We did not extract data on *leprogenic odontodysplasia*, but three of the studies included in our review described other cases, namely: a child aged 9-11 years from the St Mary Magdalen leprosy hospital in England (61), a juvenile aged 13-19 years from Denmark (48), and a child aged 11-12 years from Sigtuna, Sweden (88).” citing:**

**48. Matos VMJ, Santos AL. Leprogenic odontodysplasia: new evidence from the St. Jørgen’s medieval leprosarium cemetery (Odense, Denmark). Anthropol Sci. 2013;121(1):43–7.**

**61. Taylor GM, Tucker K, Butler R, Pike AWG, Lewis J, Roffey S, et al. Detection and Strain Typing of Ancient Mycobacterium leprae from a Medieval Leprosy Hospital. Gordon SV, editor. PLoS ONE. 2013 Apr 30;8(4):e62406.**

**88. Kjellström A. Possible cases of leprosy and tuberculosis in medieval Sigtuna, Sweden. Int J Osteoarchaeol. 2010 May;22(3):261–83.**

**134. Danielsen K. Odontodysplasia leprosa in Danish mediaeval skeletons. Tandlaegebladet. 1970 Jun;74(6):603–25.**

Genomic studies:

1. On p20 the authors state that 5 previous papers have looked for evidence of *M. lepromatosis* in genomic studies. In fact, an additional group looked and this is detailed in your main text reference 106, Mendum et al, 2018.

**We have corrected this omission.**

2. SNP type 3. This genotype is mentioned a few times within the main text. E.g. Abstract, p20, p23, This category has come to be recognized as a broad group within the Monot typing scheme, which originally described strains as belonging to SNP types 1A to 4P. Type 3 includes the oldest lineage 3K, itself sub-divided into 3K0 and 3K1, as well as 3M, now seen as nearer/basal to SNP-type 4 strains and type 3I-1, responsible for the majority of European cases in the mediaeval period and an ancestor to the 31-1 and 3I-2 types present in southern USA states (Truman et al., N. Engl. J. Med. 364, 1626, 2011). This reviewer suggests that where known, the subtype is provided in the text to provide better context; predominantly this is likely to be 3I. Similarly, the most commonly identified SNP-type 2 strain found has been 2F.

**We have added a breakdown of the SNP subtypes to the text, namely: “Of the 73 skeletons analysed, SNP type 1 was identified in one, SNP subtype 2F in 11 (15.1%), SNP type 3 was identified in 59 (80.8%), SNP types 3Q and 4 each in one (Supplementary File, Appendices). Of the 59 SNP type 3, 13 were subtype 3I, 18 were 3I-1, 11 were 3K, two were 3L and two were 3M”.**

In the concluding remarks on p29, the authors mention the importance of integrating genomic studies into future research. This is undoubtedly true, but broader biomolecular approaches may also grow in importance and provide alternative means of studying the mycobacterium. For example, mycolipids, proteins and peptides from *M. leprae*.

**We have added a mention of these other approaches, namely “the integration of genetics and, potentially, biomolecular approaches such as those based on mycolipids, proteins and peptides, will be essential..”**

Minor points:

1. Leprosaria (found on p2, p3, p10, p26, p27, p28) is usually italicized by convention to denote its Latin origins.

**All occurrences amended.**

2. “Leprosy presents a spectrum of skin and nerve clinical presentations”. This could be rephrased to better convey the intended meaning with corrected grammar.

**Sentence deleted because first sentence of paragraph had already referred to leprosy affecting skin and nerves.**

B). Supplementary Information (Appendices). xlsx file.

The following points need attention.

1. Data extraction tab, columns BT & BU. Spelling of Skulls.

**Corrected.**

2. Genomic References included Tab. “Synopsis”. Entry 3. The 2G genotyping of Economou’s paper was later corrected to 2F, in line with findings from other groups. This correction is published in JAS 40 (6), page 2867.

**We have added a footnote to the synopsis indicating “* Subtype changed from 2G to 2F in Economou et al, Corrigendum to “Ancient-DNA reveals an Asian type of Mycobacterium leprae in medieval Scandinavia” [J. Archaeol. Sci. 40 (1) (2013) 465–470], *Journal of Archaeological Science*, Volume 40, Issue 6, 2013, Page 2867”. Our review had used the correct subtype in Figure 7.**

3. QA Ratings (genomic) Tab. Entry 23/24, Roffey 2017. It is surprising to see this assessed as 00 for biomolecular analysis when this reference successfully typed 9 SNP and 3 VNTR loci to show this was a type 2F. DNA preservation was later shown to be good enough for WGS which confirmed this genotype. Details in reference 60. Perhaps this Roffey MS has been confused with Roffey, 2012 ?

**This is indeed what appears to have happened. We have corrected the quality rating for this study.**

4. Included References (Genomic) Tab. “Synopses” missing from Monot et al, 2009 and Taylor et al, 2018. Also, type 3I-1 found in Entry 21 (see Main text comment above).

**We feel that this synopsis column is not particularly helpful, given that the data extracted from each included study are reported in ‘Data extraction’ (genomic)’. We have deleted it.**

5. QA Ratings Tab, Item 6 Biomolecular Analysis. Many of the publications here do not offer genomic analysis yet have been assessed as 00 by the two independent reviewers. It would be clearer if these are flagged as either ND (not determined) or NA (not available). These publications include Blau, 2005; Baker 2014; Buckley 2008 etc.

**This is a fair point and, with hindsight, we would probably have omitted this item from the non-genomic study quality rating. Given that we do not want to make a post hoc change and that the inclusion or omission of this item makes no difference to the key aspect of our quality rating namely, excluding a small number of studies deemed to be of poor quality in terms of paleopathological methods, we will leave unchanged but bear in mind for future.**

6. Data Extraction Tab. Article ID 11 (Cole, 2022) of Appendices shows ? for total skeletons at the site. Having checked with one of the excavators, I can offer that the skeleton total excavated and analyzed to date is 125, as mentioned in Discussion in J. Med. Microbiol. 2024; 73: 001806.

**We thank the reviewer for this information and we have updated the cell accordingly.**

**Reviewer #2**

Summary: This systematic review presents a comprehensive analysis of leprosy in archaeological skeletons, synthesizing data on skeletal changes and genomic findings to explore the historical trajectory of the disease. The study provides valuable insights into the paleopathology of leprosy, including the frequency and distribution of Rhinomaxillary Syndrome (RMS) and post-cranial bone changes (PCBC). The manuscript is well-structured, methodologically sound, and contributes significantly to our understanding of leprosy’s historical impact. With minor revisions incorporating a One Health perspective and a more nuanced discussion of the decline hypothesis, the paper will be well-suited for publication. The revisions will enhance the interdisciplinary relevance of the study and align it with contemporary approaches to infectious disease history and epidemiology.

**We thank the reviewer for these encouraging remarks. We have addressed the points regarding One Health and the decline hypothesis as detailed below.**

Strengths:

1. Comprehensive Scope: The systematic review effectively synthesizes data from multiple archaeological studies across different regions and time periods.

2. Robust Methodology: The inclusion criteria, data extraction, and quality assessment procedures are clearly defined and rigorously applied.

3. Genetic Analysis Integration: The discussion on Mycobacterium leprae genotypes strengthens the argument regarding leprosy’s historical spread and decline.

4. Clarity of Presentation: The figures and tables are well-organized, enhancing the readability and interpretability of results.

**No comments to address.**

Proposed revisions:

1. Incorporating a One Health Perspective:

The discussion on the decline of leprosy in medieval Europe primarily focuses on immunological, epidemiological, and environmental factors. However, it lacks consideration of the broader One Health framework, which integrates human, animal, and environmental health interactions.

Suggested Revision: Expand the discussion to consider how zoonotic reservoirs, changes in human-animal interactions, and environmental shifts may have influenced the disease’s decline. For instance, studies suggest that other mycobacterial species present in the environment may have played a role in cross-immunity.

**We have expanded the paragraph in which we discuss the ‘decline in Europe’ hypotheses to incorporate references to cross-immunity (current gaps in knowledge and non-tuberculous mycobacterial species) and to other historical factors and we have extended our ‘Leprosy outside Europe’ with a discussion of how evidence from paleopathological research can inform One Health approaches to the control of Hansen’s disease in the present day (see responses to suggested revisions #2 and #3 below).**

2. Clarification of the Decline Hypothesis:

The current hypothesis emphasizes tuberculosis-leprosy co-infection, climate change, and improved living conditions as key factors in the decline of leprosy.

Suggested Revision: While these factors are valid, it would be beneficial to explore the role of urbanization, changing human demographics, and potential shifts in animal host populations that could have influenced transmission dynamics.

**We have expanded the paragraph in which we discuss the ‘decline in Europe’ hypotheses to incorporate references to cross-immunity (current gaps in knowledge and non-tuberculous mycobacterial species) and to other historical factors, namely “the prevalence of leprosy may have declined due to temporal changes in immune status influenced by bacterial exposure (129), but this hypothesis is complicated by gaps in our contemporary understanding of cross-immunity (137), including with other non-tuberculous mycobacterial species in the environment (138), and by the impact of other historical factors such as urbanization (139) and population movements (140). Testing such hypotheses is constrained by the small numbers of geographically and temporally dispersed remains for paleopathological research, as characterised in our review” citing:**

**137. Crespo F, White J, Roberts C. Revisiting the tuberculosis and leprosy cross-immunity hypothesis: Expanding the dialogue between immunology and paleopathology. Int J Paleopathol. 2019 Sep;26:37–47.**

**138. Turankar et al. Association of non-tuberculous mycobacteria with Mycobacterium leprae in environment of leprosy endemic regions in India. Infect Genet Evol J Mol Epidemiol Evol Genet Infect Dis. 2019 Aug;72:191–8.**

**139. Kelmelis KS, Pedersen DD. Impact of urbanization on tuberculosis and leprosy prevalence in medieval Denmark. Anthropol Anz Ber Uber Biol-Anthropol Lit. 2019 Jun 24;76(2):149–66.**

**140. Donoghue HD. Tuberculosis and leprosy associated with historical human population m ovements in Europe and beyond - an overview based on mycobacterial anc ient DNA. Ann Hum Biol. 2019 Mar;46(2):120–8.**

3. Contextualizing the Findings with Modern Epidemiology:

The discussion draws valuable parallels between historical and modern leprosy cases. However, additional references to contemporary One Health approaches in leprosy control would strengthen this connection.

Suggested Revision: Briefly mention current One Health initiatives that address zoonotic transmission of leprosy (e.g., armadillos in the Americas as reservoirs of M. leprae) and how similar dynamics might have played a role in historical contexts.

**We have extended our ‘Leprosy outside Europe’ with a discussion of how evidence from paleopathological research can inform One Health approaches to the control of Hansen’s disease in the present day: “One aspect of paleopathological research of relevance to the present day is the role of animal and environmental reservoirs of *M. leprae* (and *M. lepromatosis*) and the potential for zoonotic transmission to introduce and sustain infection in human populations (143). In present day South America, particularly in Brazil where Hansen’s disease remains endemic, *M. leprae* is prevalent in armadillos, and human contact through hunting and consumption is common, there is a compelling argument for adopting a One Health approach to Hansen’s disease (144), whilst further evidence is needed to assess the zoonotic potential of *M. lepromatosis* in the region (145). Genetic evidence from medieval Europe reinforces these links, showing close relationships between ancient *M. leprae* strains from Iberia and England and those isolated from modern red squirrels. Moreover, strains from the Late Medieval period are genetically similar to those now circulating in humans and armadillos in the Americas (146). These findings underscore how palaeopathological research clarifies historical transmission patterns that remain relevant today, supporting integrated, cross-disciplinary strategies to control leprosy in endemic settings” citing:**

**143. Ploemacher T, Faber WR, Menke H, Rutten V, Pieters T. Reservoirs and transmission routes of leprosy; A systematic review. PLoS Negl Trop Dis. 2020 Apr;14(4):e0008276.**

**144. Deps P, Rosa PS. One Health and Hansen’s disease in Brazil. PLoS Negl Trop Dis. 2021 May 27;15(5):e0009398.**

**145. Deps P, Collin SM. Mycobacterium lepromatosis as a Second Agent of Hansen’s Disease. Front Microbiol. 2021 Sep 10;12:698588.**

**146. Pfrengle S, Neukamm J, Guellil M, Keller M, Molak M, Avanzi C, et al. Mycobacterium leprae diversity and population dynamics in medieval Europe from novel ancient genomes. BMC Biol. 2021 Oct 5;19(1):220.**

4. Minor Edits for Clarity and Consistency:

Ensure consistency in the use of terminology, particularly in references to ‘possible’ and ‘probable’ RMS.

**We have checked the manuscript throughout and corrected any inconsistencies.**

Some sections could benefit from minor grammatical improvements for readability. This also applies to the styling of the references.

**We have checked the manuscript throughout for readability and made improvements.**

5. Selection of references is only partly accounted for:

There is a discrepancy between the 86 in the systematic review and the overall 134 references. 40% of the references are not accounted for.

**Of the 134 total references, the first 39 were cited in the Introduction and Methods, followed by 86 references to the studies included for data extraction in the actual review, and a then a further 9 citations to support our Discussion. In revising our manuscript, we have added two more references to the Introduction and eleven more to the Discussion in response to reviewers’ comments to give a total of 147 references. Note that Methods is now final section hence, all references have been re-ordered.**

**Reviewer #3**

This paper aims to bring together all of the evidence from published leprosy cases from across the globe with a view to better understanding its evolutionary history, temporal geographic spread and changes in lesion presentation. It does this through a literature review and some reassessment of images. The paper is generally well written and within the scope of interest of the journal readership. That being said, I have some major reservations about the work that need addressing before it can be accepted for publication. I hope that these comments are useful for the authors so that they make get their work published. A major problem with this paper is that there is a lack of critical thought about the palaeopathological data, its limitations and meaning. While I think what the authors suggest from the data is probably correct, there are multiple explanations for what they have observed and these should be addressed. In addition, there is no specific research question(s), or aims which means the results and discussion is a bit haphazard and unfocused. A hypothesis or two would help here.

**We thank the reviewer for their critical review of our study and its reporting. We have attempted to address their comments in full, and we think that the revised manuscript is much improved. Our objectives were to describe (summarize) the paleopathological data using methods (formal systematic review) that have not been applied previously to this subject. In investigating variation over time and correlations, we are not testing pre-specified hypotheses, and the nature of the data are such that the appropriate statistical tests are necessarily basic. We have made a large number of revisions, as described above, including engaging in more detail with the limitations and potential modern day utility of paleopathological data, and we think that these have greatly improved the manuscript.**

A significant limitation is that the sample is pre-selected for having leprosy. That is, the lesions were usually distinct enough that someone was confident that the individual could be diagnosed with leprosy in the first place. In sticking only to cases that they can be confident in recognising then we are really only looking at the most severe manifestation of the disease. Given we miss the cases of leprosy without skeletal lesions, or discard the ones with less obvious lesions, it is very difficult to compare what we have with modern data generally. Furthermore, when talking about disease presence/prevalence, we also have to be very careful (discussion page 22). The presence of leprosaria in particular are problematic because they may be acting to concentrate cases giving a perception that the disease was becoming increasingly common. To understand this, we would need to know far more about leprosaria and what their ‘catchments’ may have been as well as more precise dating of burials - this does not exist yet. This would then need to be considered with the context of increasing population size through time. Yes, there were more cases in the late medieval period, but the population was also much larger than it had been before!

**We would counter that a pre-selected paleopathological population of people with more severe manifestations of leprosy can still be informative on a ‘like-for-like’ basis, and we have extended our paragraph to provide more detail as to how “Direct examination of bone changes in paleopathology specimens can improve understanding of clinical manifestations of M. leprae infection in living persons affected by Hansen’s disease (122). For example, we previously used computed tomography (CT) scans to assess the prevalence of RMS among elderly former patients of a ‘colony’ hospital in Brazil (123). We also used CT imaging combined with otorhinolaryngological examinations to assess maxillofacial and nasal manifestations of leprosy in current patients (124). Findings from these studies suggest that clinical protocols for Hansen’s disease could be extended to include otorhinolaryngological evaluation, supported by imaging where necessary, to improve the assessment and overall care of persons affected by Hansen’s disease.” We assert that “This systematic review synthesizes evidence from palaeopathological studies, characterising cranial and post-cranial bone alterations associated with leprosy, serving as a reference point for clinical research and practice today.” We have extended our ‘Leprosy outside Europe’ section with a discussion of how evidence from paleopathological research can inform One Health approaches to the control of Hansen’s disease in the present day (in response to another review comment).**

**We have replaced the term ‘prevalent’ (with ‘more frequent’) where this might be misconstrued as having an epidemiological (population-level) meaning and changed ‘supporting hypotheses’ to ‘exploring hypotheses’ [for the decline of leprosy in Europe].**

**We have added to the text of our Results a breakdown of RMS by leprosaria *vs*. non-*leprosaria* sites, and discussed these as follows: “Although the location and type of archaeological site might be expected to influence the types of bone alterations in remains, we found equal proportions (15%) of skulls without RMS from *leprosaria* and from general cemeteries or other burial sites. This observation challenges the assumption that *leprosaria* served as focal destinations and final resting places for people in advanced disease stages, who would presumably exhibit distinctly identifiable facial deformities (122,123) or that individuals within leprosaria necessarily experienced prolonged survival, allowing more time for such deformities to develop. However, we also recognise that the entire paleopathological population in our review is pre-selected for the more severe forms of leprosy that result in visible bone changes, regardless of site of exhumation, which explains the overall frequent occurrence of RMS in our study.”**

This preselection for diagnostic leprosy cases is also problematic for understanding lesion patterns too. Those lesions that are diagnostic for leprosy are going to be inflated in comparison to other types. As such, I am not surprised that RMS was observed in 80% of cases given this is likely the clue that got them accepted as a case and then published! Additionally, the increase in RMS over time in England could well be because those in leprosaria were getting support so lived longer and developed more pronounced lesions. At Winchester, special feeding equipment was found. Furthermore, for the less diagnostic and non-specific lesions, we need to know how common they are they in the populations in general, and what can be attributed to the disease. What would be more interesting would be an aged-matched comparison of lesions from individuals in leprosaria with those outside leprosaria as this may tell us about efficacy of treatment and life outcomes of people with the condition.

**We have added some analysis of leprosaria vs. non-leprosaria data and acknowledged the preselection issue as described in our response to the preceding comment. We have also incorporated the reviewer’s suggestion for future analysis into the penultimate paragraph of our Discussion, namely: “Newer imaging modalities such as micro-CT may allow for deeper characterisation of bone alterations and provide a way of confirming leprosy diagnosis in fragmented remains (147). These techniques may also support age-matched comparisons between individuals from leprosaria and those from general burial sites, enabling researchers to explore the effects of institutional care, treatment access, and disease progression on skeletal pathology.”**

This paper needs to discuss the current issues with inter and intra-observer error that exists between palaeopathologists. There is no detailed methodology for recording post cranial changes at present so what someone might score as present, another might not. This has been the significant factor that has really prevented such a large-scale study such as that presented here. It is simply very difficult to reliably compare data. How can you overcome this problem?

**We feel that these issues are beyond the scope of our review and not something that we can overcome, other than in rating the quality of each included study (an aspect of our review that we would revisit in future, given that it did not specifically rate a primary study’s mitigations for or reporting of intra/inter-observer error). We have added a mention of this issue to our limitations, namely: “The lack of a standardized investigative protocol for identifying leprosy-related bone changes means that the secondary data summarised in our review may be susceptible to intra- and inter-observer error made by the original researchers.”**

In many parts of the discussion, the authors draw attention to a number of issues or themes surrounding leprosy evolution/history yet they have not linked this to new data that they have produced. For example, the link between TB and leprosy and the decline of the latter, or the distributions of the strains. They need to explicitly show how their data adds new information to these discussions beyond what is already discussed in the references and citations or remove it from the text.

**We have expanded the paragraph in which we discuss the ‘decline in Europe’ hypotheses to incorporate references to cross-immunity (current gaps in knowledge and non-tuberculous mycobacterial species) and to other historical factors, namely “the prevalence of leprosy may have declined due to temporal changes in immune status influenced by bacterial exposure (129), but this hypothesis is complicated by gaps in our contemporary understanding of cross-immunity (137), including with other non-tuberculous mycobacterial species in the environment (138), and by the impact of other historical factors such as urbanization (139) and population movements (140). Testing such hypotheses is constrained by the small numbers of geographically and temporally dispersed remains for paleopathological research, as characterised in our review” citing:**

**137. Crespo F, White J, Roberts C. Revisiting the tuberculosis and leprosy cross-immunity hypothesis: Expanding the dialogue between immunology and paleopathology. Int J Paleopathol. 2019 Sep;26:37–47.**

**138. Turankar et al. Association of non-tuberculous mycobacteria with Mycobacterium leprae in environment of leprosy endemic regions in India. Infect Genet Evol J Mol Epidemiol Evol Genet Infect Dis. 2019 Aug;72:191–8.**

**139. Kelmelis KS, Pedersen DD. Impact of urbanization on tuberculosis and leprosy prevalence in medieval Denmark. Anthropol Anz Ber Uber Biol-Anthropol Lit. 2019 Jun 24;76(2):149–66.**

**140. Donoghue HD. Tuberculosis and leprosy associated with historical human population m ovements in Europe and beyond - an overview based on mycobacterial anc ient DNA. Ann Hum Biol. 2019 Mar;46(2):120–8.**
