# Supplementary material for: Leprosy in skeletons from archaeological sites: A systematic review
Source: PLoS Negl Trop Dis. 2025 Aug 19;19(8):e0013374. doi: 10.1371/journal.pntd.0013374 (PMC12364322; doi:10.1371/journal.pntd.0013374)
Supplement: Attachment — Submitted filename: Responses_to_Reviewers_auresp_2.docx [file pntd.0013374.s007.docx]

**Response to Reviewers’ Comments**

**Journal requirements**

1) We have noticed that you have uploaded Supporting Information files, but you have not included a complete list of legends. Please add a full list of legends for your Supporting Information file "PRISMA_2020_checklist.docx " after the references list.

**We have added a legend for the PRISMA checklist.**

**A. Methods**

Reviewer #1: (No Response).

Reviewer #2: The methods are adequately described.

Reviewer #3: This work is much improved and I thank the authors for taking my comments on board.

I would suggest that they are clearer about whether the lesions scored for the post crania can be directly connected to leprosy or not. over 50% of femurs being affected is high, and importantly much higher than the tibia or fibula. This is highly irregular as the femur is rarely affected. Is this a typo?

**We thank the reviewer for this important observation. The finding that 54% (25/46) of femurs were reported as affected does indeed appear atypical when compared to the lower proportions of involvement seen in tibias (14%) and fibulas (15%). This is not a typographical error but reflects the actual frequencies reported in the included studies. However, we agree that this warrants careful interpretation, as femoral involvement is generally considered uncommon in leprosy.**

**There is limited evidence in the paleopathological literature suggesting that the femur is frequently affected in leprosy. Typically, skeletal lesions most often involve the small bones of the hands and feet, the nasal region, and the tibiae due to their subcutaneous location and proximity to cooler areas of the body, where *Mycobacterium leprae* tends to localize (Ortner, 2003; Manchester, 1984). Femoral lesions are less commonly reported.**

**Given the relatively small number of femurs examined (n=46), the high percentage (54%) may reflect sampling bias, preservation bias, or reporting bias in the available osteoarchaeological studies. For instance, femora may be better preserved or more frequently recovered than smaller bones in certain burial contexts, potentially leading to overrepresentation. Alternatively, studies may have focused more prominently on femoral lesions when they occurred, given their relative rarity, leading to a visibility bias.**

**We have added a paragraph to our Discussion (page 25) to acknowledge this anomaly and we have added a statement cautioning against overinterpretation of the finding due to the small sample size and possible biases. Unfortunately, to the best of our knowledge, no published source provides a systematic estimate of expected femoral involvement in leprosy cases in archaeological remains, which limits our ability to cite counterbalancing evidence.**

**B. Results**

Reviewer #1: (No Response).

Reviewer #2: here is a mistake in the closure date of the Batavia leprosarium in the table: it should be 1896. The results are clearly and completely presented

**We have corrected the closure date.**

Reviewer #3: (No Response).

**C. Conclusions**

Reviewer #1: (No Response).

Reviewer #2: I have two remarks with regard to the conclusions:

1. Try to include the results of the following study:

Urban C, Blom AA, Avanzi C, Walker-Meikle K, Warren AK, White-Iribhogbe K, Turle R, Marter P, Dawson-Hobbis H, Roffey S, Inskip SA, Schuenemann VJ. Ancient Mycobacterium leprae genome reveals medieval English red squirrels as animal leprosy host. Curr Biol. 2024 May 20;34(10):2221-2230.e8. doi: 10.1016/j.cub.2024.04.006.

**We have added this citation to our Discussion (page 30) where we state that “Phylogenetic evidence from medieval Europe reinforces these links, with a study from Winchester in England showing a close relationship between ancient *M. leprae* squirrel and human strains and suggesting that *M. leprae* circulated in non-human hosts in the Middle Ages (147)”.**

2. In the twentieth century there still is endemic leprosy in Spain and Portugal: See:

Suárez-García I, Gómez-Barroso D, Fine PEM. Autochthonous leprosy in Spain: Has the transmission of Mycobacterium leprae stopped? PLoS Negl Trop Dis. 2020 Sep 16;14(9):e0008611. doi: 10.1371/journal.pntd.0008611.

Ferreira PM, Rato IR, Rigor J, Mota M. Hansen's disease - a forgotten disease? JRSM Open. 2021 Aug 30;12(8):20542704211035995. doi: 10.1177/20542704211035995

**We have revised our Introduction, changing “was largely eliminated” to “had mostly disappeared” and appending “although a small number of autochthonous cases have occurred in southern Europe in the 21^st^ Century” citing Suárez-García and Ferreira.**

Reviewer #3: (No Response).

**D. Editorial and Data Presentation Modifications**

Reviewer #1: (No Response).

Reviewer #2: No modifications needed.

Reviewer #3: (No Response).

**E. Summary and General Comments**

Reviewer #1: I consider the authors have now revised their manuscript, taking into account the points raised in review or reasonably rebutted individual issues.

Reviewer #2: Okay apart from the false statement that leprosy in Europe was eliminated in the 19th century.

**We have revised our Introduction, changing “was largely eliminated” to “had mostly disappeared” and appending “although a small number of autochthonous cases have occurred in southern Europe in the 20th Century” citing Suárez-García and Ferreira.**

Reviewer #3: (No Response).
